# The genome sequence of common reed, *Phragmites australis* (Cav.) Steud. (Poaceae)

**DOI:** 10.12688/wellcomeopenres.23143.1

**Published:** 2024-10-11

**Authors:** Maarten J. M. Christenhusz, Michael F. Fay, Royal Botanic Gardens Kew Genome Acquisition Lab, Plant Genome Sizing collective, Darwin Tree of Life Barcoding collective, Wellcome Sanger Institute Tree of Life Management, Samples and Laboratory team, Wellcome Sanger Institute Scientific Operations: Sequencing Operations, Wellcome Sanger Institute Tree of Life Core Informatics team, Tree of Life Core Informatics collective, Darwin Tree of Life Consortium

**Affiliations:** 1 Royal Botanic Gardens Kew, Richmond, England, UK; 2 Curtin University, Perth, Western Australia, Australia

**Keywords:** Phragmites australis, common reed, genome sequence, chromosomal, Poales

## Abstract

We present a genome assembly from an individual *Phragmites australis* (the common reed; Streptophyta; Magnoliopsida; Poales; Poaceae). The genome sequence has a total length of 848.70 megabases. Most of the assembly is scaffolded into 24 chromosomal pseudomolecules, supporting the specimen being an allotetraploid (2 *n* = 4 *x* = 48). The three mitochondrial assemblies had lengths of 304.58, 92.24, and 76.54 kilobases and the plastid genome assembly had a length of 137.67 kilobases. Gene annotation of this assembly on Ensembl identified 47,513 protein-coding genes.

## Species taxonomy

Eukaryota; Viridiplantae; Streptophyta; Streptophytina; Embryophyta; Tracheophyta; Euphyllophyta; Spermatophyta; Magnoliopsida; Mesangiospermae; Liliopsida; Petrosaviidae; commelinids; Poales; Poaceae; PACMAD clade; Arundinoideae; Molinieae; Molininae; *Phragmites*; *Phragmites australis* (Cav.) Steud. (NCBI:txid29695).

## Background

Common reed, *Phragmites australis* (Cav.) Steud., is a globally widespread grass species that is found across its range in wet habitats, although it is absent from the lowland wet tropics ( POWO, 2024). It was originally described as *Arundo phragmites* by Linnaeus 1753, based on European material, but when placed in the genus *Phragmites*, the oldest name is described from Australia (Botany Bay, coll. Luis Née) by Cavanilles ( Cavanilles, 1799).

*Phragmites australis* is a rhizomatous grass, with nodose, hollow stems and leaf blades with a central fold and a clasping sheath. The tall stems are topped with plumes of bisexual, wind-pollinated flowers. It usually grows on water edges and in swamps, on fresh or brackish water (up to 16% salt; Ostendorp, 1993), and can be a pioneer species on newly exposed shores.

The reeds are traditionally used as a roofing material across north-western Europe, especially in Britain and the Netherlands ( Köbbing *et al.*, 2013). Indeed, there is evidence that the reed has been used for roof thatching along the North and Baltic Sea coastlines ever since the last ice age ( Iital *et al.*, 2012; Schaatke, 1992). In the past it had a wide range of applications from manufacturing boats to providing forage. Reed is a cheap, abundant and sustainable material, and annual harvesting increases the vigour and vitality of reed stands ( Huhta, 2009). Current possibilities for reed utilisation can be divided into industrial, energy, agricultural and water treatment uses (see Köbbing *et al.*, 2013 for an overview). In addition, reeds can also be used as an insulation material, garden fencing, walls, pulp and paper, and polymerisation for textile or plastic ( Bundesministerium für Wirtschaft und Technologie, 2012; Chivu, 1968). They can also be harvested for renewable biofuel ( ELP & Ash, 2010; Wichmann & Wichtmann, 2009). In agriculture, reeds can be used as fodder or composted for mulch ( Huhta, 2009).

*Phragmites australis* can be harvested from the wild, but it is also actively planted for use as a natural filter for polluted water ( Kusler & Kentula, 1990; Sarafraz *et al.*, 2009; Vymazal, 2010). The use for water treatment is especially effective when the reed beds are harvested for biofuel production, so that pollutants are removed from the environment.

Reed-dominated vegetation is host to a large community of animals, from reed warblers to harvest mice. They form an important ecological function, from cleaning water to providing shelter and forming new land.

Chromosome studies have revealed that *P. australis* is one of the most ploidy-variable species reported to date, with 10 ploidy levels based on *x* = 12 (3 *x*, 4 *x*, 6 *x*, 7 *x*, 8 *x*, 9 *x*, 12 *x*, 14 *x*, 16 *x*, 20x) reported to date ( te Beest *et al.*, 2012). In Europe, tetraploid individuals (i.e. 2 *n* = 4 *x* = 48) are reported to be the most dominant ( Clevering & Lissner, 1999). These are thought to be allotetraploids, formed through hybridisation of two diploid progenitor species which diverged >30 million years ago, although their identifies are currently unknown, and may indeed be extinct ( Clevering & Lissner, 1999; Wang *et al.*, 2024).

Two other genome assemblies for *P. australis* have recently been generated, one of these (GCA_021018715.1) to the level of contigs ( Oh *et al.*, 2022) and the other (GCA_040373225.1) to chromosomal level, comprising 24 pseudochromosomes and one B chromosome ( Wang *et al.*, 2024). The genome presented here is the current reference genome.

This high-quality genome will help with understanding the genetic diversity and can aid in developing different strains for the multiple purposes of this versatile plant.

## Genome sequence report

**Figure 1.**
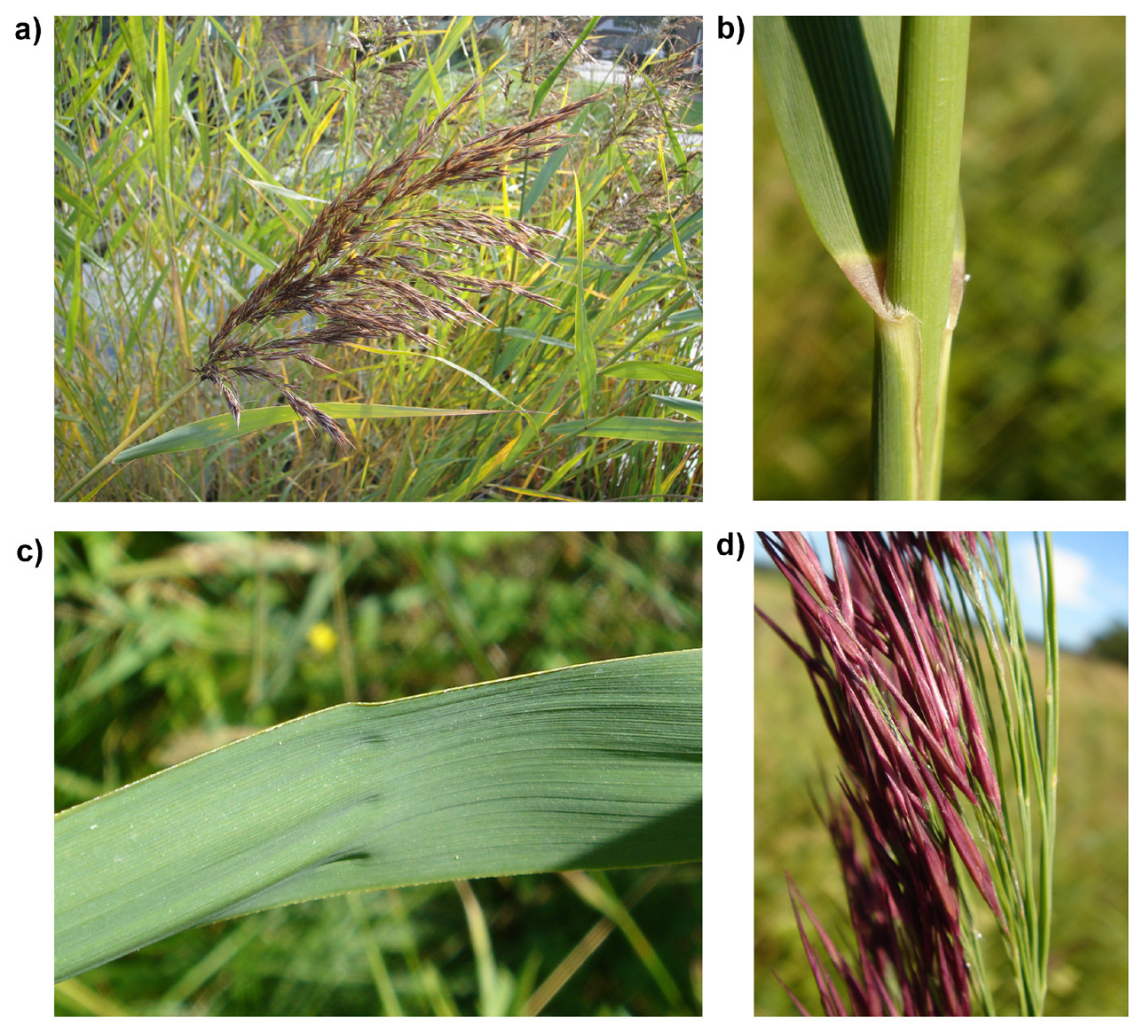
Photographs of the *Phragmites australis* (lpPhrAust1) specimen used for genome sequencing.

The genome of a specimen of *Phragmites australis* ( Figure 1) was sequenced using Pacific Biosciences single-molecule HiFi long reads, generating a total of 24.60 Gb (gigabases) from 2.60 million reads, providing approximately 26-fold coverage. Using flow cytometry, the genome size (1C-value) was estimated to be 1.08 pg, equivalent to 1,050 Mb. Primary assembly contigs were scaffolded with chromosome conformation Hi-C data, which produced 96.90 Gb from 641.75 million reads, yielding an approximate coverage of 114-fold. Specimen and sequencing information is summarised in Table 1.

**Table 1.** Specimen and sequencing data for *Phragmites australis*.

Project information			
**Study title**	Phragmites australis		
**Umbrella BioProject**	PRJEB55339		
**Species**	*Phragmites australis*		
**BioSample**	SAMEA10369659		
**NCBI taxonomy ID**	29695		
Specimen information			
**Technology**	**ToLID**	**BioSample accession**	**Organism part**
**PacBio long read sequencing**	lpPhrAust1	SAMEA10369738	leaf
**Hi-C sequencing**	lpPhrAust1	SAMEA10369738	leaf
**RNA sequencing**	lpPhrAust1	SAMEA10369738	leaf
Sequencing information			
**Platform**	**Run accession**	**Read count**	**Base count (Gb)**
**Hi-C Illumina NovaSeq 6000**	ERR10084069	6.42e+08	96.9
**PacBio Sequel IIe**	ERR10077562	2.60e+06	24.6
**RNA Illumina NovaSeq 6000**	ERR10378026	5.63e+07	8.5

**Figure 2.**
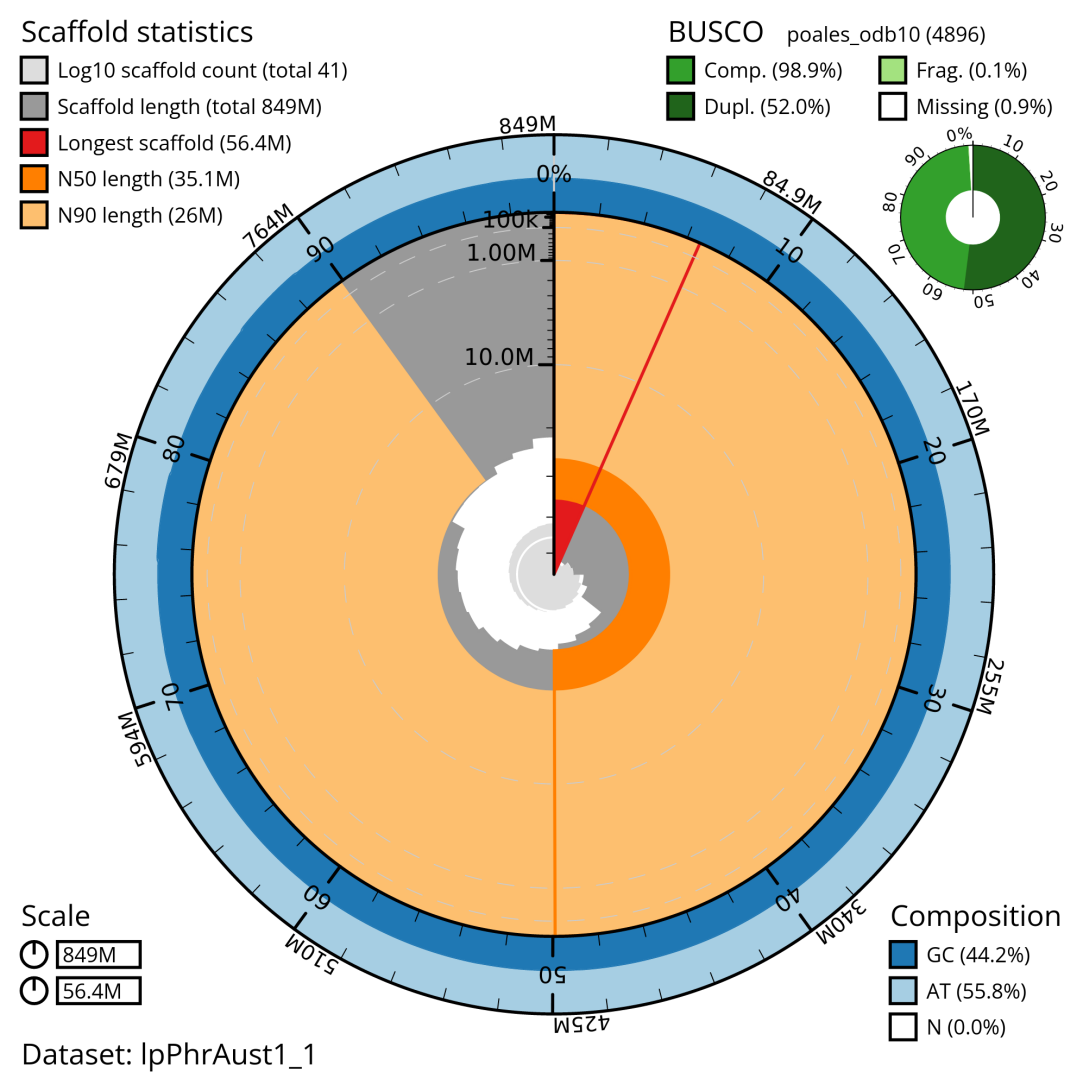
Genome assembly of *Phragmites australis*, lpPhrAust1.1: metrics. The BlobToolKit snail plot shows N50 metrics and BUSCO gene completeness. The main plot is divided into 1,000 size-ordered bins around the circumference with each bin representing 0.1% of the assembly. The distribution of scaffold lengths is shown in dark grey with the plot radius scaled to the longest scaffold present in the assembly (shown in red). Orange and pale-orange arcs show the N50 and N90 scaffold lengths (35,123,032 and 26,029,537 bp), respectively. The pale grey spiral shows the cumulative scaffold count on a log scale with white scale lines showing successive orders of magnitude. The blue and pale-blue area around the outside of the plot shows the distribution of GC, AT and N percentages in the same bins as the inner plot. A summary of complete, fragmented, duplicated and missing BUSCO genes in the poales_odb10 set is shown in the top right. An interactive version of this figure is available at https://blobtoolkit.genomehubs.org/view/lpPhrAust1_1/dataset/lpPhrAust1_1/snail.

**Figure 3.**
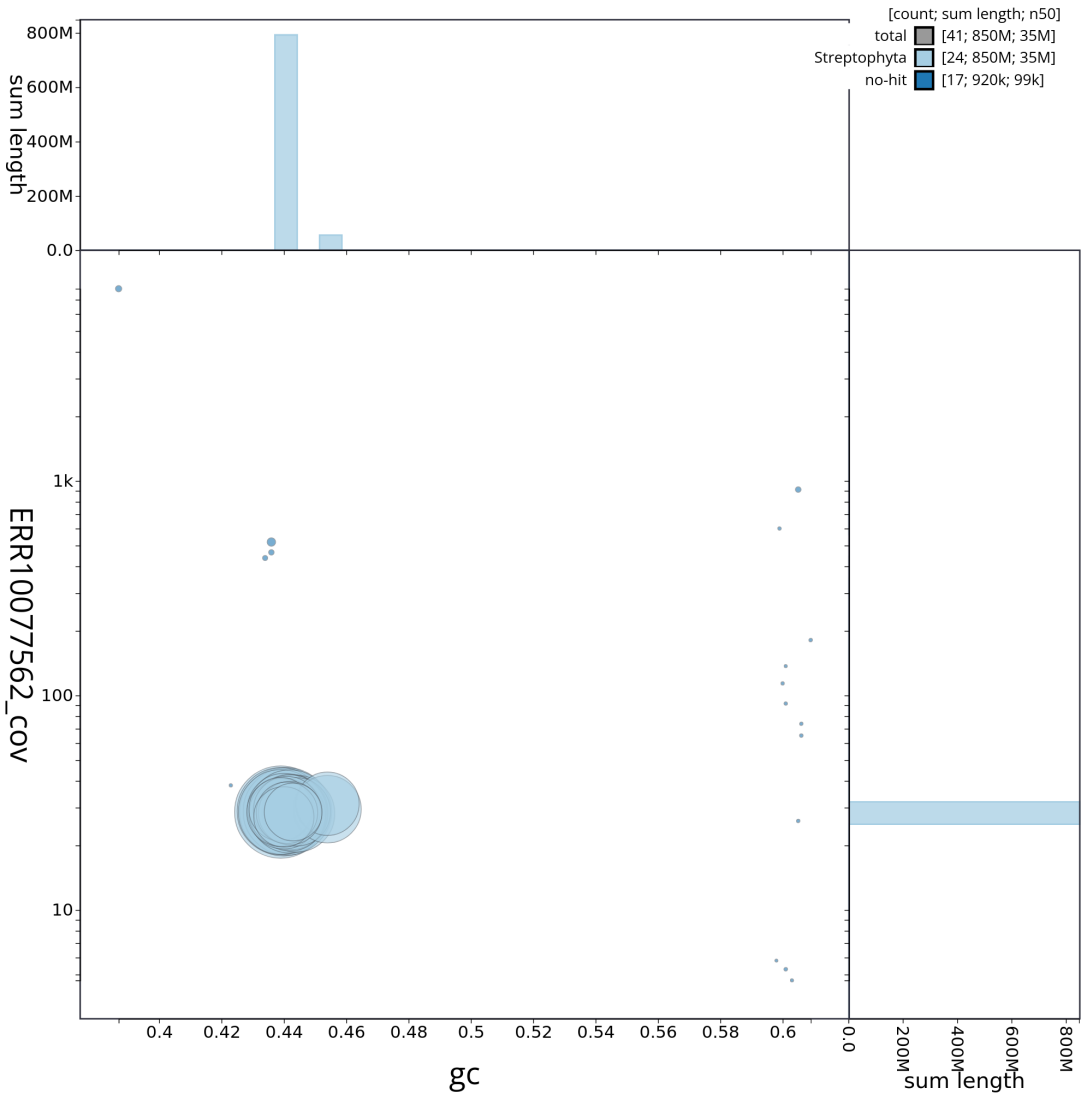
Genome assembly of *Phragmites australis*, lpPhrAust1.1: BlobToolKit GC-coverage plot. Scaffolds are coloured by phylum. Circles are sized in proportion to scaffold length. Histograms show the distribution of scaffold length sum along each axis. An interactive version of this figure is available at https://blobtoolkit.genomehubs.org/view/lpPhrAust1_1/dataset/lpPhrAust1_1/blob.

**Figure 4.**
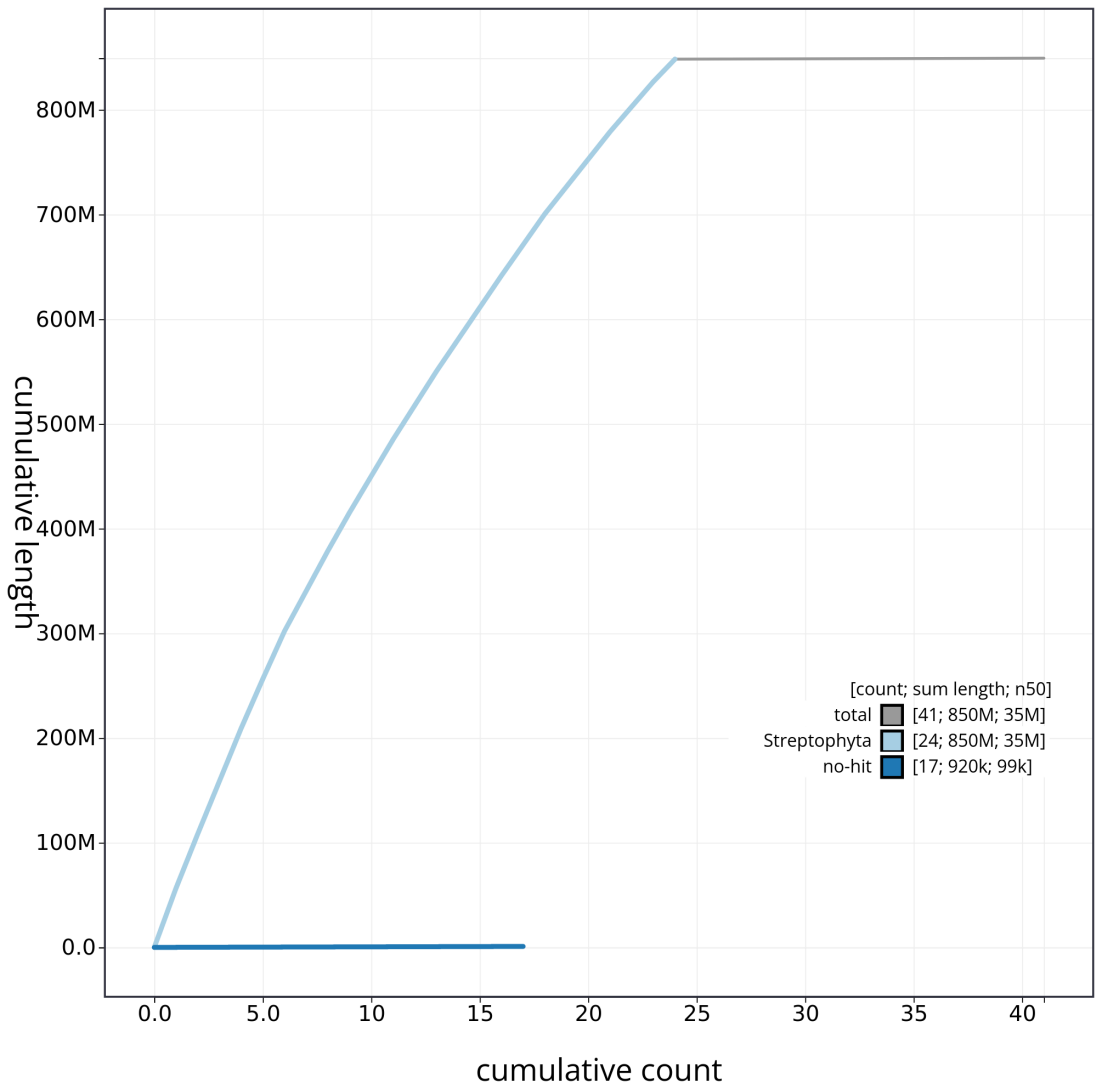
Genome assembly of *Phragmites australis*, lpPhrAust1.1: BlobToolKit cumulative sequence plot. The grey line shows cumulative length for all scaffolds. Coloured lines show cumulative lengths of scaffolds assigned to each phylum using the buscogenes taxrule. An interactive version of this figure is available at https://blobtoolkit.genomehubs.org/view/lpPhrAust1_1/dataset/lpPhrAust1_1/cumulative.

**Figure 5.**
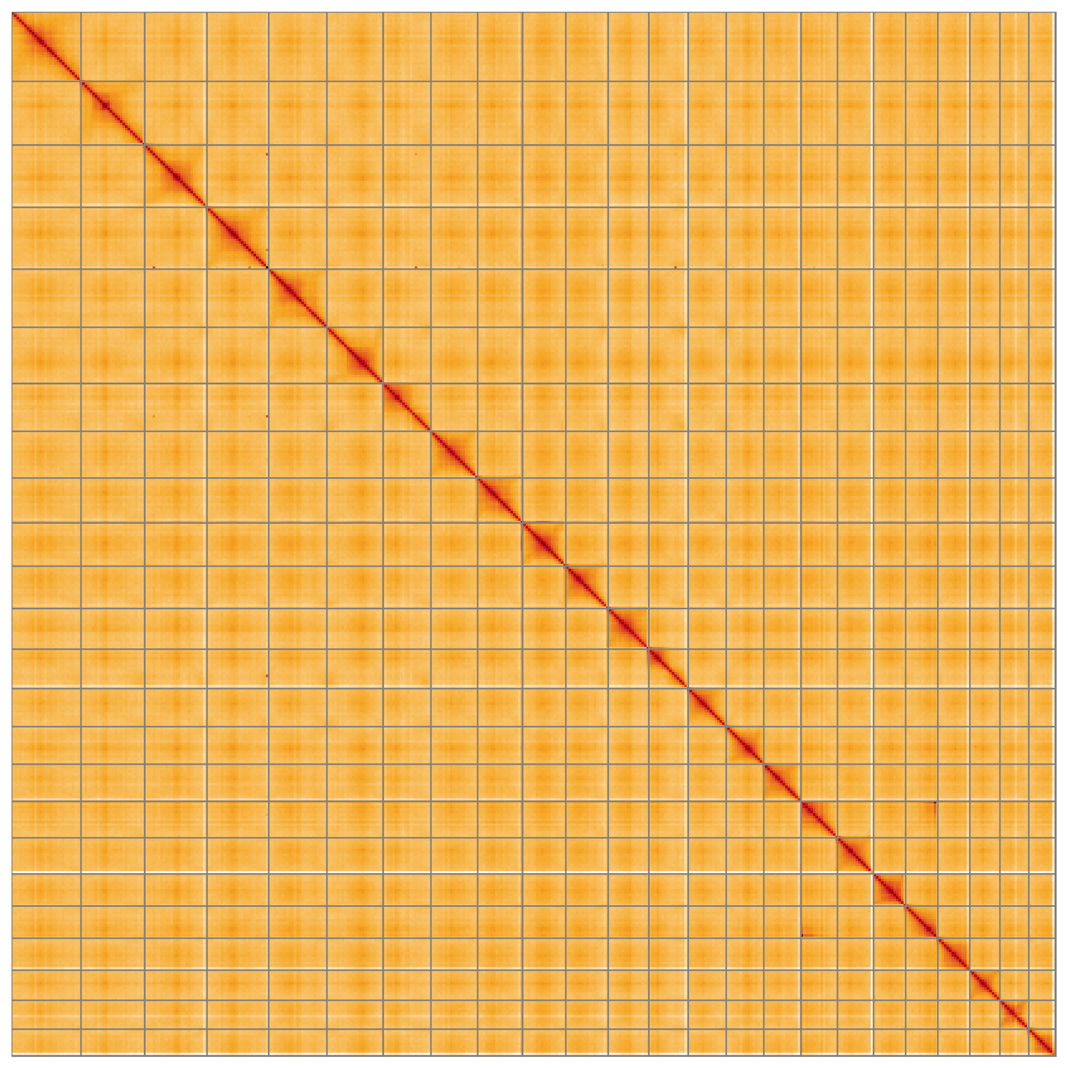
Genome assembly of *Phragmites australis*, lpPhrAust1.1: Hi-C contact map of the lpPhrAust1.1 assembly, visualised using HiGlass. Chromosomes are shown in order of size from left to right and top to bottom. An interactive version of this figure may be viewed at https://genome-note-higlass.tol.sanger.ac.uk/l/?d=MglbMUeiT9ehQAPpyumgfA.

Manual assembly curation corrected 56 missing joins or mis-joins, reducing the scaffold number by 51.76%, and also decreasing the scaffold N50 by 23.06%. The final assembly has a total length of 848.70 Mb in 37 sequence scaffolds with a scaffold N50 of 35.1 Mb ( Table 2) with 59 gaps. The snail plot in Figure 2 provides a summary of the assembly statistics, while the distribution of assembly scaffolds on GC proportion and coverage is shown in Figure 3. The cumulative assembly plot in Figure 4 shows curves for subsets of scaffolds assigned to different phyla. Most (99.89%) of the assembly sequence was assigned to 24 chromosomal-level scaffolds. Chromosome-scale scaffolds confirmed by the Hi-C data are named in order of size ( Figure 5; Table 3). Given that there are 24 unique scaffolds, the data support that the specimen is an allotetraploid (2 *n* = 4 *x* = 48). A B chromosome was not assembled. Contigs corresponding to alternate haplotypes have also been deposited.

**Table 2.** Genome assembly data for *Phragmites australis*, lpPhrAust1.1.

Genome assembly		
Assembly name	lpPhrAust1.1	
Assembly accession	GCA_958298935.1	
*Accession of alternate haplotype*	*GCA_958298975.1*	
Span (Mb)	848.70	
Number of contigs	100	
Contig N50 length (Mb)	30.9	
Number of scaffolds	37	
Scaffold N50 length (Mb)	35.1	
Longest scaffold (Mb)	56.43	
Assembly metrics *		*Benchmark*
Consensus quality (QV)	66.0	*≥ 50*
*k*-mer completeness	100.0%	*≥ 95%*
BUSCO **	C:98.9%[S:46.9%,D:52.0%], F:0.1%,M:0.9%,n:4,896	*C ≥ 95%*
Percentage of assembly mapped to chromosomes	99.89%	*≥ 95%*
Organelles	Mitochondrial genome: of 304.58, 92.24, and 76.54 kb; plastid genome: 137.67 kb	*complete single* *alleles*
Genome annotation at Ensembl		
Number of protein-coding genes	47,513	
Number of non-coding genes	12,985	
Number of gene transcripts	88,861	

* Assembly metric benchmarks are adapted from column VGP-2020 of “Table 1: Proposed standards and metrics for defining genome assembly quality” from Rhie *et al.* (2021). ** BUSCO scores based on the poales_odb10 BUSCO set using version 5.4.3. C = complete [S = single copy, D = duplicated], F = fragmented, M = missing, n = number of orthologues in comparison. A full set of BUSCO scores is available at https://blobtoolkit.genomehubs.org/view/lpPhrAust1_1/dataset/lpPhrAust1_1/busco.

**Table 3.** Chromosomal pseudomolecules in the genome assembly of *Phragmites australis*, lpPhrAust1.

INSDC accession	Name	Length (Mb)	GC%
OY282612.1	1	56.43	44.0
OY282613.1	2	51.79	44.0
OY282614.1	3	50.62	44.0
OY282615.1	4	50.2	44.0
OY282616.1	5	47.27	44.0
OY282617.1	6	45.65	44.0
OY282618.1	7	38.87	44.5
OY282619.1	8	37.92	44.0
OY282620.1	9	36.59	44.0
OY282621.1	10	35.12	44.0
OY282622.1	11	34.44	44.0
OY282623.1	12	33.03	44.0
OY282624.1	13	32.06	44.0
OY282625.1	14	30.88	44.0
OY282626.1	15	30.5	44.5
OY282627.1	16	30.16	44.0
OY282628.1	17	29.71	45.5
OY282629.1	18	29.25	44.0
OY282630.1	19	26.18	44.0
OY282631.1	20	26.11	45.5
OY282632.1	21	26.03	44.0
OY282633.1	22	24.46	44.0
OY282634.1	23	23.46	44.0
OY282635.1	24	21.61	44.5
OY282639.1	Pltd	0.14	38.5
OY282636.1	MT1	0.3	43.5
OY282637.1	MT2	0.09	43.5
OY282638.1	MT3	0.08	43.5

The mitochondrial and plastid genomes were also assembled and can be found as contigs within the multifasta file of the genome submission, and as sequences with their own accession numbers.

The estimated Quality Value (QV) of the final assembly is 66.0 with *k*-mer completeness of 100.0%, and the assembly has a BUSCO v5.3.2 completeness of 98.9% (single = 46.9%, duplicated = 52.0%), using the poales_odb10 reference set ( *n* = 4,896).

Metadata for specimens, BOLD barcode results, spectra estimates, sequencing runs, contaminants and pre-curation assembly statistics are given at https://links.tol.sanger.ac.uk/species/29695.

## Genome annotation report

The *Phragmites australis* genome assembly (GCA_958298935.1) was annotated at the European Bioinformatics Institute (EBI) on Ensembl Rapid Release. The resulting annotation includes 88,861 transcribed mRNAs from 47,513 protein-coding and 12,985 non-coding genes ( Table 2; https://rapid.ensembl.org/Phragmites_australis_GCA_958298935.1/Info/Index). The average transcript length is 3,454.91. There are 1.47 coding transcripts per gene and 4.97 exons per transcript.

## Methods

### Sample acquisition, DNA barcoding and genome size estimation

A *Phragmites australis* (specimen ID KDTOL10363, ToLID lpPhrAust1) was collected from Kingston upon Thames, Surrey, UK (latitude 51.44, longitude –0.33) on 2021-08-17. The specimen was collected and identified by Maarten Christenhusz (Royal Botanic Gardens, Kew) and preserved by dry-freezing at –80 °C. The herbarium voucher associated with the sequenced plant has been deposited in the herbarium of RBG Kew (K).

The initial species identification was verified by an additional DNA barcoding process according to the framework developed by Twyford *et al*. (2024). Part of the plant specimen was preserved in silica gel desiccant. A DNA extraction from the dried plant was amplified by PCR for standard barcode markers, with the amplicons sequenced and compared to public sequence databases including GenBank and the Barcode of Life Database (BOLD). The barcode sequences for this specimen are openly available on BOLD ( Ratnasingham & Hebert, 2007). Following whole genome sequence generation, DNA barcodes were also used alongside the initial barcoding data for sample tracking through the genome production pipeline at the Wellcome Sanger Institute (WSI) ( Twyford *et al.*, 2024). The standard operating procedures for the Darwin Tree of Life barcoding have been deposited on protocols.io ( Beasley *et al.*, 2023).

The genome size was estimated by flow cytometry using the fluorochrome propidium iodide and following the ‘one-step’ method as outlined in Pellicer *et al.* (2021). For this species, the General Purpose Buffer (GPB) supplemented with 3% PVP and 0.08% (v/v) beta-mercaptoethanol was used for isolation of nuclei ( Loureiro *et al.*, 2007), and the internal calibration standard was *Petroselinum crispum* ‘Champion Moss Curled’ with an assumed 1C-value of 2,200 Mb ( Obermayer *et al.*, 2002).

### Nucleic acid extraction

The workflow for high molecular weight (HMW) DNA extraction at the WSI Tree of Life Core Laboratory includes a sequence of core procedures: sample preparation and homogenisation, DNA extraction, fragmentation and purification. Detailed protocols are available on protocols.io ( Denton *et al.*, 2023). The lpPhrAust1 sample was weighed and dissected on dry ice ( Jay *et al.*, 2023) and leaf tissue was cryogenically disrupted using the Covaris cryoPREP ^®^ Automated Dry Pulverizer ( Narváez-Gómez *et al.*, 2023).

HMW DNA was extracted using the Automated Plant MagAttract v2 protocol ( Todorovic *et al.*, 2023). HMW DNA was sheared into an average fragment size of 12–20 kb in a Megaruptor 3 system ( Bates *et al.*, 2023). Sheared DNA was purified by solid-phase reversible immobilisation, using AMPure PB beads to eliminate shorter fragments and concentrate the DNA ( Strickland *et al.*, 2023). The concentration of the sheared and purified DNA was assessed using a Nanodrop spectrophotometer and Qubit Fluorometer and Qubit dsDNA High Sensitivity Assay kit. Fragment size distribution was evaluated by running the sample on the FemtoPulse system.

RNA was extracted from leaf tissue of lpPhrAust1 in the Tree of Life Core Laboratory at the WSI using the RNA Extraction: Automated MagMax™ *mir*Vana protocol ( do Amaral *et al.*, 2023). The RNA concentration was assessed using a Nanodrop spectrophotometer and a Qubit Fluorometer using the Qubit RNA Broad-Range Assay kit. Analysis of the integrity of the RNA was done using the Agilent RNA 6000 Pico Kit and Eukaryotic Total RNA assay.

### Hi-C preparation

Hi-C data were generated from leaf tissue of the lpPhrAust1 sample, using the Arima-HiC v2 kit. Tissue was finely ground using cryoPREP, and then subjected to nuclei isolation using a modified protocol of the Qiagen QProteome Kit. After isolation, the nuclei were fixed, and the DNA crosslinked using a 37% formaldehyde solution. The crosslinked DNA was then digested using the restriction enzyme master mix. The 5’-overhangs were then filled in and labelled with biotinylated nucleotides and proximally ligated. An overnight incubation was carried out for enzymes to digest remaining proteins and for crosslinks to reverse. A clean up was performed with SPRIselect beads prior to library preparation. DNA concentration was quantified using the Qubit Fluorometer v2.0 and Qubit HS Assay Kit according to the manufacturer’s instructions.

### Library preparation and sequencing

Library preparation and sequencing were performed at the WSI Scientific Operations core. Pacific Biosciences HiFi circular consensus DNA sequencing libraries were constructed according to the manufacturers’ instructions. Libraries were prepared using the PacBio Express Template Preparation Kit v2.0 (Pacific Biosciences, California, USA) as per the manufacturer's instructions. The kit includes the reagents required for removal of single-strand overhangs, DNA damage repair, end repair/A-tailing, adapter ligation, and nuclease treatment. Library preparation also included a library purification step using 0.8X AMPure PB beads (Pacific Biosciences, California, USA) and size selection step to remove templates <5 kb using AMPure PB modified SPRI. DNA concentration was quantified using the Qubit Fluorometer v2.0 and Qubit HS Assay Kit and the final library fragment size analysis was carried out using the Agilent Femto Pulse Automated Pulsed Field CE Instrument and gDNA 55 kb BAC analysis kit. Samples were sequenced using the Sequel IIe system (Pacific Biosciences, California, USA). The concentration of the library loaded onto the Sequel IIe was within the manufacturer's recommended loading concentration range of 40–100 pM. The SMRT link software, a PacBio web-based end-to-end workflow manager, was used to set-up and monitor the run, as well as perform primary and secondary analysis of the data upon completion.

For Hi-C library preparation, DNA was fragmented to a size of 400 to 600 bp using a Covaris E220 sonicator. The DNA was then enriched, barcoded, and amplified using the NEBNext Ultra II DNA Library Prep Kit, following manufacturers’ instructions. The Hi-C sequencing was performed using paired-end sequencing with a read length of 150 bp on an Illumina NovaSeq 6000.

Poly(A) RNA-Seq libraries were constructed using the NEB Ultra II RNA Library Prep kit, following manufacturer’s instructions, and RNA sequencing was performed on the Illumina NovaSeq 6000 instrument.

### Genome assembly, curation and evaluation

***Assembly***

The HiFi reads were first assembled using Hifiasm ( Cheng *et al.*, 2021) with the --primary option. Haplotypic duplications were identified and removed using purge_dups ( Guan *et al.*, 2020). The Hi-C reads were mapped to the primary contigs using bwa-mem2 ( Vasimuddin *et al.*, 2019). The contigs were further scaffolded using the provided Hi-C data ( Rao *et al.*, 2014) in YaHS ( Zhou *et al.*, 2023) using the --break option. The scaffolded assemblies were evaluated using Gfastats ( Formenti *et al.*, 2022), BUSCO ( Manni *et al.*, 2021) and MERQURY.FK ( Rhie *et al.*, 2020).

The organelle genomes were assembled using OATK ( Zhou, 2023).

***Curation***

The assembly was decontaminated using the Assembly Screen for Cobionts and Contaminants (ASCC) pipeline (article in preparation). Manual curation was primarily conducted using PretextView ( Harry, 2022), with additional insights provided by JBrowse2 ( Diesh *et al.*, 2023) and HiGlass ( Kerpedjiev *et al.*, 2018). Scaffolds were visually inspected and corrected as described by Howe *et al*. (2021). Any identified contamination, missed joins, and mis-joins were corrected, and duplicate sequences were tagged and removed. The process is documented at https://gitlab.com/wtsi-grit/rapid-curation (article in preparation).

***Evaluation of final assembly***

A Hi-C map for the final assembly was produced using bwa-mem2 ( Vasimuddin *et al.*, 2019) in the Cooler file format ( Abdennur & Mirny, 2020). To assess the assembly metrics, the *k*-mer completeness and QV consensus quality values were calculated in Merqury ( Rhie *et al.*, 2020). This work was done using the “sanger-tol/readmapping” ( Surana *et al.*, 2023a) and “sanger-tol/genomenote” ( Surana *et al.*, 2023b) pipelines. The genome readmapping pipelines were developed using the nf-core tooling ( Ewels *et al.*, 2020), use MultiQC ( Ewels *et al.*, 2016), and make extensive use of the Conda package manager, the Bioconda initiative ( Grüning *et al.*, 2018), the Biocontainers infrastructure ( da Veiga Leprevost *et al.*, 2017), and the Docker ( Merkel, 2014) and Singularity ( Kurtzer *et al.*, 2017) containerisation solutions. The genome was analysed within the BlobToolKit environment ( Challis *et al.*, 2020) and BUSCO scores ( Manni *et al.*, 2021) were calculated.

Table 4 contains a list of relevant software tool versions and sources.

**Table 4.** Software tools: versions and sources.

Software tool	Version	Source
BlobToolKit	4.2.1	https://github.com/blobtoolkit/blobtoolkit
BUSCO	5.3.2	https://gitlab.com/ezlab/busco
bwa-mem2	2.2.1	https://github.com/bwa-mem2/bwa-mem2
Cooler	0.8.11	https://github.com/open2c/cooler
Gfastats	1.3.6	https://github.com/vgl-hub/gfastats
Hifiasm	0.16.1-r375	https://github.com/chhylp123/hifiasm
HiGlass	1.11.6	https://github.com/higlass/higlass
Merqury	MerquryFK	https://github.com/thegenemyers/MERQURY.FK
OATK	0.2	https://github.com/c-zhou/oatk
PretextView	0.2	https://github.com/wtsi-hpag/PretextView
purge_dups	1.2.3	https://github.com/dfguan/purge_dups
sanger-tol/genomenote	v1.0	https://github.com/sanger-tol/genomenote
sanger-tol/readmapping	1.1.0	https://github.com/sanger-tol/readmapping/tree/1.1.0
YaHS	yahs-1.1.91eebc2	https://github.com/c-zhou/yahs

### Wellcome Sanger Institute – Legal and Governance

The materials that have contributed to this genome note have been supplied by a Darwin Tree of Life Partner. The submission of materials by a Darwin Tree of Life Partner is subject to the **‘Darwin Tree of Life Project Sampling Code of Practice’**, which can be found in full on the Darwin Tree of Life website here. By agreeing with and signing up to the Sampling Code of Practice, the Darwin Tree of Life Partner agrees they will meet the legal and ethical requirements and standards set out within this document in respect of all samples acquired for, and supplied to, the Darwin Tree of Life Project.

Further, the Wellcome Sanger Institute employs a process whereby due diligence is carried out proportionate to the nature of the materials themselves, and the circumstances under which they have been/are to be collected and provided for use. The purpose of this is to address and mitigate any potential legal and/or ethical implications of receipt and use of the materials as part of the research project, and to ensure that in doing so we align with best practice wherever possible. The overarching areas of consideration are:

•   Ethical review of provenance and sourcing of the material

•   Legality of collection, transfer and use (national and international) 

Each transfer of samples is further undertaken according to a Research Collaboration Agreement or Material Transfer Agreement entered into by the Darwin Tree of Life Partner, Genome Research Limited (operating as the Wellcome Sanger Institute), and in some circumstances other Darwin Tree of Life collaborators.

## Data Availability

European Nucleotide Archive: *Phragmites australis*. Accession number PRJEB55339; https://identifiers.org/ena.embl/PRJEB55339 ( Wellcome Sanger Institute, 2023). The genome sequence is released openly for reuse. The *Phragmites australis* genome sequencing initiative is part of the Darwin Tree of Life (DToL) project. All raw sequence data and the assembly have been deposited in INSDC databases. Raw data and assembly accession identifiers are reported in Table 1.

## Author information

Members of the Royal Botanic Gardens Kew Genome Acquisition Lab are listed here: https://doi.org/10.5281/zenodo.12625079.

Members of the Plant Genome Sizing collective are listed here: https://doi.org/10.5281/zenodo.7994306.

Members of the Darwin Tree of Life Barcoding collective are listed here: https://doi.org/10.5281/zenodo.12158331.

Members of the Wellcome Sanger Institute Tree of Life Management, Samples and Laboratory team are listed here: https://doi.org/10.5281/zenodo.12162482.

Members of Wellcome Sanger Institute Scientific Operations: Sequencing Operations are listed here: https://doi.org/10.5281/zenodo.12165051.

Members of the Wellcome Sanger Institute Tree of Life Core Informatics team are listed here: https://doi.org/10.5281/zenodo.12160324.

Members of the Tree of Life Core Informatics collective are listed here: https://doi.org/10.5281/zenodo.12205391.

Members of the Darwin Tree of Life Consortium are listed here: https://doi.org/10.5281/zenodo.4783558.
