# Supplementary material for: The genome sequence of common reed, Phragmites australis (Cav.) Steud. (Poaceae)
Source: Wellcome Open Res. 2024 Oct 11;9:577. [Version 1] doi: 10.12688/wellcomeopenres.23143.1 (PMC11549543; doi:10.12688/wellcomeopenres.23143.1)
Supplement: Peer review history

# Reviewer report: Reviewer response for version 1

**DOI:** 10.21956/wellcomeopenres.25485.r108835

**Published:** 2024-11-08

**Authors:** Cui Wang

**Affiliations:** 1 University of Helsinki, Helsinki, Finland

The authors present a chromosome-level genome assembly of *Phragmites australis* in their manuscript, “The genome sequence of common reed, *Phragmites australis* (Cav.) Steud. (Poaceae).” This genome has been selected as a Refseq entry in the NCBI database, and is a high quality genome assembly representing European population of *P. australis*. The dataset is sufficient, the results are well described, and the data is openly accessible, thus this work adds good value on research of genome evolution within large species complexes. While I recommend this manuscript for indexing, a few points need to be addressed:

1. Flow cytometry results suggest the genome size of the sample is around 1,050 Mb, while the final assembly is 848.70 Mb, suggesting a discrepancy of roughly 200Mb (nearly 20% of the genome) potentially missing. This difference may result from inaccuracies in the flow cytometry measurement. Please address this in the text.
2. Page 3 “Manual assembly curation corrected 56 missing joins or mis-joins”. Please revise the sentence, missing joins and mis-joins are two different issues. The current phrasing implies they are the same. Please clarify.
3. Page 3 “B chromosome was not assembled.” What does this mean? It's possible that your individual doesn't have a B chromosome.
4. The gene annotation. Besides the Ensemble database, this version of genome has been also annotated using NCBI’s pipeline. According to NCBI, the annotated number of protein coding gene is 38444, while Ensembl pipeline annotated 47,513 protein coding genes, with nearly 9,000 genes different. Could you clarify the reason for this discrepancy and indicate which annotation is preferred?

Are sufficient details of methods and materials provided to allow replication by others?

Yes

Is the rationale for creating the dataset(s) clearly described?

Yes

Are the datasets clearly presented in a useable and accessible format?

Yes

Are the protocols appropriate and is the work technically sound?

Yes

Reviewer Expertise:

Genomics, evolutionary genetics, ecology, transcriptomics

I confirm that I have read this submission and believe that I have an appropriate level of expertise to confirm that it is of an acceptable scientific standard.

# Reviewer report: Reviewer response for version 1

**DOI:** 10.21956/wellcomeopenres.25485.r104750

**Published:** 2024-10-24

**Authors:** Peng-Fei Ma

**Affiliations:** 1 Germplasm Bank of Wild Species & Yunnan Key Laboratory of Crop Wild Relatives Omics, Kunming Institute of Botany, Chinese Academy of Sciences, Kunming, China

In this paper, the authors presented a high-quality genome assembly of tetraploid common reed. This is a globally widespread species and has long been used as roofing material. In addition, this species has variable ploidy levels, posing challenges for genome sequencing. The genome assembly will be helpful for studying the evolution of this important species. In the sentence 'Manual assembly curation corrected ...., and also decreasing the scaffold N50 by 23.06%'., why reducing the scaffold number while with N50 length decreased?

Are sufficient details of methods and materials provided to allow replication by others?

Yes

Is the rationale for creating the dataset(s) clearly described?

Yes

Are the datasets clearly presented in a useable and accessible format?

Yes

Are the protocols appropriate and is the work technically sound?

Yes

Reviewer Expertise:

Plant phylogeny, genomics and evolution.

I confirm that I have read this submission and believe that I have an appropriate level of expertise to confirm that it is of an acceptable scientific standard.
